# The Role of Spatial Frequency Information in Face Classification by Race

**DOI:** 10.3389/fpsyg.2017.01152

**Published:** 2017-08-24

**Authors:** Guoping Zhang, Zeyao Wang, Jie Wu, Lun Zhao

**Affiliations:** ^1^The China Academy of Corporate Governance and Business School, Nankai University Tianjin, China; ^2^The Center for Brain and Cognitive Sciences and Department of Psychology, Peking University Beijing, China; ^3^Eli Broad College of Business, Michigan State University, East Lansing MI, USA; ^4^Institute of Psychology and Behavior, Tianjin Normal University Beijing, China; ^5^Institute of Brain Research, Beijing Yiran Sunny Technology Co., Ltd. Beijing, China

**Keywords:** faces’ race, face classification, other-race advantage, spatial frequency

## Abstract

It was found that face classification by race is more quickly for other-race than own-race faces (other-race classification advantage, ORCA). Controlling the spatial frequencies of face images, the current study investigated the perceptual processing differences based on spatial frequencies between own-race and other-race faces that might account for the ORCA. Regardless of the races of the observers, the own-race faces were classified faster and more accurately for broad-band faces than for both lower and higher spatial frequency (SF) faces, whereas, although other-race faces were classified less accurately for higher SF than for either broad-band or lower SF faces, there was no difference between broad-band and lower SF conditions of other-race faces. Although it was not evident for higher SF condition, the ORCA was more evident for lower SF than that for broad-band faces. The present data indicate that global/configural information is needed for subordinate race categorization of faces and that an important source of ORCA is application of global/configural computations by default while categorizing an own-race face but not while categorizing an other-race face.

## Introduction

It is well known that human adults can identify better the age, gender, expression, and identity of own-race faces than of other-race faces, i.e., “the other-race effect” (see for a review, Meissner and Brigham, 2001). On the other hand, there was evidence that the catigorization of faces by race is more quickly for other- than own-race faces, i.e., “the other-race classification advantage” (ORCA). For example, Caucasian participants classify Black or Asian faces faster than Caucasian faces (Valentine and Endo, 1992; Levin, 1996; Caldara et al., 2004; Zhao and Bentin, 2008, 2011).

Generally, humans use several perceptual strategies while processing a face at different levels. At the basic-level, the characteristic global structure of human faces, i.e., the recruitment of global/holistic processes, is what distinguishes faces from non-face objects. The specific global structure of faces refers to the first-order relations comprising of two eyes located above the nose and the mouth and on both sides of a vertical axis including the nose and the mouth (Maurer et al., 2002). On the basis of the fact that all normal human faces share the first-order relations, identifying a face at the within-category individuation level indeed relies on a deeper analysis of both the face components such as the eyes, the nose, and the mouth (“feature analysis”) and the computation of spatial-relations between the inner components of faces (“configural analysis”; Maurer et al., 2002). Cross-culture studies have shown that configural as well as feature analysis were better for own-race than other-race faces due to the lack of experience with other-race faces (e.g., Tanaka et al., 2004; Rhodes et al., 2006; Hayward et al., 2008). Although some studies investigated the relationship between face featural and configural processing and other-race effect (e.g., Rhodes et al., 1989; Tanaka et al., 2004; Michel et al., 2006), few studies were conducted for ORCA. Since the second-order configuration is considered as a major source of identification of individual faces (e.g., Searcy and Bartlett, 1996), it is acceptable that categorizing faces by race does not rely on configural computation. Supporting this view, there was evidence that Caucasian participants classified other-race faces faster than own-race faces although face inversion slowed down the overall classification time (Levin, 2000), suggesting that differential configural processing among races cannot account for ORCA. In contrast, in one recent cross-race study, Zhao and Bentin (2011) found that the ORCA was similarly robust for full faces and for face parts and larger for faces with distorted configuration, suggesting that the configural processing is a major source of the ORCA.

The spatial frequency (SF) scales of facial information are generally used to categorizing faces. The image with high spatial frequencies (HSF) represents the fine-scale details of the original image, while the low spatial frequencies (LSF) retain the large-scale global shape of visual formation. Recent studies supported the associations between HSF and local processing and between LSF and configural processing by manipulating spatial frequencies corresponding to featural or configural information (e.g., Goffaux et al., 2005; Goffaux and Rossion, 2006) and hence, spatial filtering might be efficient to assess the necessity of details in race categorization of faces. A large number of reports have explored the role of spatial frequencies in face recognition, but, to our knowledge, little have directly investigated the use of spatial frequencies in race categorization of faces. In one recent study about the effect of special frequency information on subordinate categorization, Harel and Bentin (2009) found that subordinate categorization of faces by race was not impaired for facial images with LSF but was significantly slower and less accurate for faces when LSF were filtered out, suggesting that the global and configural information might be essential for race categorization. However, ORCA was not found in that study at any of the SF scales and therefore, the association between SF and ORCA should be concluded with caution. To our knowledge there are no studies that examined directly the importance of different SF for ORCA, which would be done by comparing the use of SF in race categorization within and across races.

In this study, the accuracy and RT measures were used to explore the consequences of spatial-frequency manipulations on the categorization of own and other races by race. Chinese and Caucasian participants classified faces with broad band spatial frequency (BSF), lower spatial frequency (LSF), and higher spatial frequency (HSF) features as Chinese or Caucasian. If the global/configural information is necessary for the race categorization of faces, the ORCA should be evident for LSF than HSF conditions (c.f., Zhao and Bentin, 2011).

## Materials and Methods

### Participants

The participants were 20 Chinese undergraduates (10 female, 20–26 years) and 17 Caucasian students (8 female, 21–28 years; no more than one year in China) from Nankai University in China. All participants were right handed based on self-report, with normal or corrected to normal visual acuity and no history of psychiatric or neurological disorders. They signed an informed consent to participate to this study as requested by the Institutional Review Board (IRB) of the Nankai University and were paid for participation.

Either before or after the experiment, participants completed one self-report questionnaire including five items regarding other-race contact (Voci and Hewstone, 2003; Walker et al., 2008). Item (i) asked, ‘How many Caucasian (for Chinese participants) or Chinese (for Caucasian participants) people do you know very well?’ with the answer choices: Up to 2, Up to 5, Up to 8, Up to 12 and More than 12. Items 2–4 used the following scale: strongly agree (5 point), sort of agree (4 point), not sure (3 point), sort of disagree (2 point), strongly disagree (1 point) and were worded as: (i) ‘I often talk to Caucasian (for Chinese participants) or Chinese (for Caucasian participants) people in college’, (ii) ‘I often see Caucasian (for Chinese participants) or Chinese (for Caucasian participants) people outside of college’, (iii) ‘I often hang out with Caucasian (for Chinese participants) or Chinese (for Caucasian participants) people’ and (iv) ‘I often see Caucasian (for Chinese participants) or Chinese (for Caucasian participants) people at social events I attend’. Overall, the participants exhibited below mid-point social-contact for other-race faces, 2.4 ± 0.6 and 2.3 ± 0.7 for Chinese and Caucasian participants, respectively (*p* = 0.68), reflecting relatively little experience with faces from the other race.

### Stimuli

**FIGURE 1:**
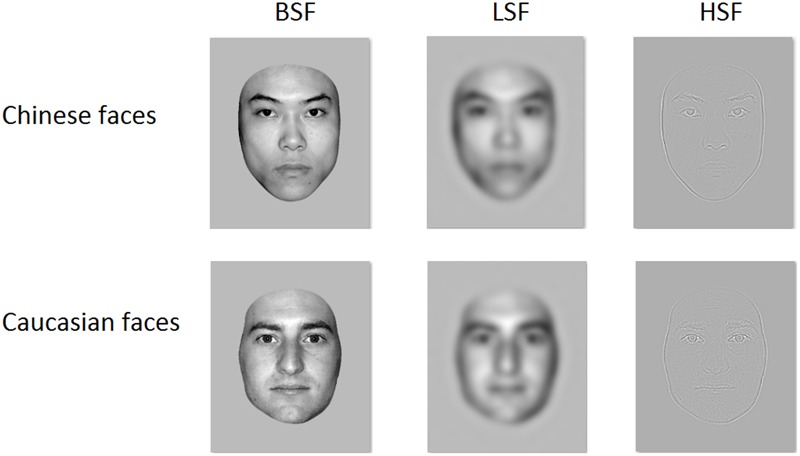
Examples of Chinese and Caucasian stimuli, respectively.

Stimuli consisted of 54 gray-scale photographs of young Chinese (27 male, 27 female; 20–30 years old) and 54 young Caucasian (27 male, 27 female; 20–30 years old; all Caucasian faces are Germans) faces. All faces were unfamiliar to the participants. These photographs were used to form three stimulus types, broad-band (BB), lower spatial-frequency (LSF), and higher spatial-frequency (HSF) faces (**Figure 1**). The original BB faces were spatially filtered using a Butterworth filter with an exponent of 4. The LSF and HSF filter cutoff corners were 1 cycle/degree (∼10 cycles/image) and 6.5 cycles/degree (65 cycles/image), respectively, matching previous studies (e.g., Goffaux et al., 2003a,b; Harel and Bentin, 2009). They were showed in frontal view, with eyes aligned on the horizontal midline of the screen, equated for luminance and root mean square (RMS) contrast using Adobe Photoshop 7.0^1^, and viewed from a distance of 100 cm at a visual angle of approximately 7.9 × 6.2°.

### Design and Procedure

A mixed experimental design 2 × 2 × 3 was applied: Race-of-the-observer (Chinese vs. Caucasian) × Race-of-the-face (own-race vs. other-race) × Type-of-stimulus (BB, LSF, and HSF). The participants were seated in a dimly lit and sound-attenuated cabin and were instructed to categorize each stimulus by the race it represented and to respond to Chinese or Caucasian faces by pressing correspondingly labeled buttons on the keyboard with the left (‘Z’ key) or right index finger (‘/’ key), respectively. Speed and accuracy were equally emphasized. All stimuli were randomly presented in a mixed design with three blocks of 100 stimuli each, with a short break in between, and the labels of the response buttons (Chinese–Caucasian/ Caucasian–Chinese) were counterbalanced across the participants. Each face was presented at the center of the computer screen for 500 ms with an inter-stimulus interval (ISI) ranging randomly between 400 and 600 ms, starting after response. The participants completed one practice sequence of 24 stimuli, which were not used in the main experiment.

Both the reaction times (RTs) (from the stimulus onset) and the accuracy rates were recorded and analyzed by a Repeated Measures ANOVA (RMANOVA), with the Race-of-the-observer (Chinese, Caucasian) as a between-groups factor and the Race-of-the-face (own-race, other-race) and Type-of-stimulus (BB, LSF and HSF) as within-subjects factors. For each participant and experimental condition RTs that were more extreme than ±2SD from the mean have been excluded (less than 2%). Degrees of freedom were corrected whenever necessary using the Greenhouse–Geisser epsilon correction factor.

## Results

**FIGURE 2:**
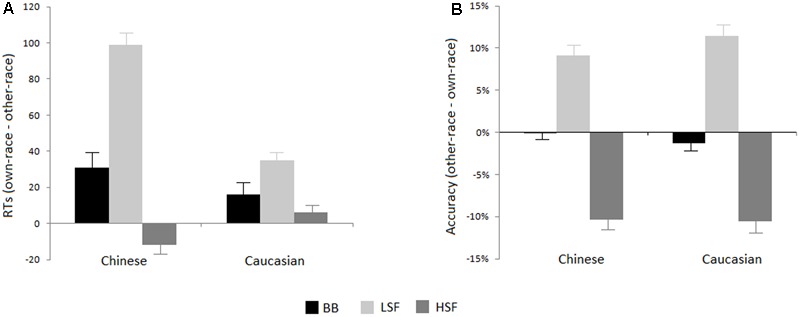
The other-race classification advantage (ORCA) of RTs **(A)** and accuracies **(B)** of own- and other-race faces for different SF conditions. More positive values indicate larger ORCA (faster or more accurate categorization of other- than own-race faces). Error bars represent standard errors of the means.

Mean RTs (to correct responses only) and accuracy for the different experimental conditions (two races of faces and three types of stimuli) are presented in **Table 1** and the ORCA effects are presented in **Figure 2**.

**Table 1:** Reaction times in ms (SD) and percentage of accuracy (SD) for own-race and other race faces at three SF scales.

Participants		Own-race faces			Other-race faces		
		BB	LSF	HSF	BB	LSF	HSF
Chinese	RTs	705 (100)	776 (172)	768 (158)	674 (99)	677 (102)	780 (190)
	Accuracy	92 (8)	81 (15)	85 (14)	92 (13)	90 (8)	75 (18)
Caucasian	RTs	714 (156)	740 (166)	780 (199)	698 (102)	705 (109)	774 (196)
	Accuracy	92 (11)	81 (14)	86 (13)	90 (9)	92 (6)	75 (19)

### Reaction Times

The Repeated Measures ANOVA showed that, overall, the main effect of Race-of-the-observer did not reach significant level [730 and 735 ms for Chinese and Caucasian participants, respectively; *F*(1,35) < 1]. Moreover, we did not find any significant interactions between the Race-of-the-observer and any other factors (*ps* > 0.1). The main effect of Race-of-the-face was significant, *F*(1,35) = 17.6, *p* < 0.001, η_p_^2^ = 0.35, indicating that categorizing other-race stimuli were faster (718 ms) than own-race stimuli (747 ms). We also found a significant main effect of Stimulus Type, *F*(2,70) = 37.67, *p* < 0.001, η_p_^2^ = 0.52, indicating that faces with BB faces were classified faster (698 ms) than LSF faces (725 ms, p < .005), which were faster than HSF (776 ms, *p* < 0.001). Importantly, we found a significant two-way interaction of Race-of-the-face ^∗^ Stimulus Type [*F*(2,70) = 7.41, *p* < 0.01; η_p_^2^ = 0.18]. Further analysis showed that ORCA was not evident at HSF condition (777 and 774 ms for other-race and own-race faces; *p* = 0.748), whereas there was a significant ORCA for BB (686 and 710 ms for other-race and own-race faces; *p* < 0.01) and LSF (691 and 758 ms for other-race and own-race faces; *p* < 0.005) faces. Indeed, the ORCA was more evident for LSF (RT_own-race_ face *minus* RT_other-race_ face: 67 ms) than that for BB faces (24 ms; *p* < 0.005). On the other hand, for own-race condition the BB faces were classified faster than faces with either LSF (*p* < 0.005) or HSF (*p* < 0.001) faces, with no difference between the latter two conditions (*p* = 0.27); for other-race condition, HSF faces were classified slower than faces with either BB (*p* < 0.001) or LSF faces (*p* < 0.001), with no difference between the latter two conditions (*p* = 0.47).

Previous study reported that the ORCA reflects primarily the difficulty to classify own-race faces as a homogeneous group rather than facilitation of classifying other-race faces (Zhao and Bentin, 2011). To further investigate this view, we conducted the correlation (Pearson) test between RTs and the size of the ORCA and found an overall significant positive correlation (Pearson) between the RTs to own-race stimuli and the size of the ORCA (*r* = 0.61, *p* < 0.01) but not between the RTs to other-race stimuli and the size of the ORCA (*r* = 0.16, *p* > 0.1).

### Accuracy

Similar to the ANOVA for RTs, for the percentage of correct responses there was no main effect of Race-of-the-observer (85.8 and 85.9% for Caucasian and Chinese participants, respectively; *F*(1,35) < 1) and there was no interaction between the Race-of-the-observer and any other factors. The ANOVA also showed that although there was no significant main effect of Race-of-face (86.1 and 85.8 % for own-race and other-race stimuli, respectively; *F*(1,35) < 1), the main effect of Type-of-stimulus was significant [*F*(2,70) = 53.78, *p* < 0.0001; η_p_^2^ = 0.61], reflecting that broad-band faces were classified more accurately (91.7%) than low-frequency faces (85.9%, *p* < 0.005), which, in turn, were classified more accurately than high-frequency faces (80.2%, *p* < 0.05). Interestingly, this effect was qualified by a two-way interaction of Type-of-stimulus ^∗^ Race-of-face [*F*(2,70) = 26.11, *p* < 0.0001; η_p_^2^ = 0.43]. Further analysis showed that for broad-band face condition, there was no significant race effect (91.9 and 91.2% for own-race and other-race faces; *p* = 0.61), whereas for low-frequency condition own-race stimuli were classified less accurately (80.8 %) than other-race stimuli (91.1%; *p* < 0.0005) and for high-frequency condition own-race stimuli were classified more accurately (85.5%) than other-race stimuli (75.0%; *p* < 0.005). On the other hand, for own-race condition, broad-band faces were classified more accurately than high-frequency stimuli (*p* < 0.03), which were classified more accurately than low-frequency stimuli (*p* < 0.05); for other-race condition, high-frequency face stimuli were classified less accurately than either broad-band faces (*p* < 0.001) or low-frequency stimuli (*p* < .001), with no difference between the latter two conditions (*p* = 0.83).

## Discussion

The current experiment explored the importance of spatial frequencies (SF) of faces for classification of Chinese and Caucasian faces by race. Overall, BB faces were categorized faster than either LSF or HSF faces. Interestingly, categorizing a face by race was highly accurate even when it reserved only either LSF isolating global/configural processing or HSF isolating the featural processing. Yet, it is noteworthy that classification accuracy for HSF faces was reduced relative to BB faces by about 12%, whereas the accuracy for LSF faces was about 6% lower than for BB faces. Therefore, it appears that the LSF is a much better cue and a major source at least for diversity between Chinese and Caucasians. Not surprisingly, race classification is best while the face with broad-band frequencies is available.

Interestingly, across the races of observers, the own-race BB faces were classified more accurately than either LSF or HSF faces, implicating that the race classification of the own-race faces relies on both global/configural and featural information. Because BB faces simply offer more information to the visual system, it is not surprisingly when all the face frequencies are available race classification is best. For other-race condition, however, although HSF faces were classified less accurately than either BB or LSF faces, there was no difference between BB and LSF conditions, indicating that categorizing faces as other-race faces mainly relies on global/configural information reflected by LSF information. Obviously, the more complex processing of own-race faces vs. other-race faces could result in the delayed response for own-race classification by race.

The present fact that both Chinese and Caucasian participants categorized other-race faces faster than own-race faces, i.e., ORCA, replicated previous studies using full faces, isolated eyes, faces with the eyes concealed, or faces with distorted configuration (Zhao and Bentin, 2008, 2011). Although there was a significant ORCA for both BB and LSF faces, the ORCA was more evident for LSF than that for BB faces, suggesting that the LSF information plays an important role for the ORCA. In one recent study about the effect of special frequency information on subordinate categorization, Harel and Bentin (2009) found that subordinate categorization of faces by race was significantly impaired when the LSF was filtered out but was not influenced when the HSF was absent, suggesting that the global/configural information might be essential for race categorization. However, ORCA was not found in that study at any of the SF scales. How could this apparent discrepancy between the two studies be reconciled? A possible answer comes considering the difference in the task demands. Whereas Harel and Bentin (2009) conducted a category verification task in which each trial started with the presentation of a category label (e.g., “Chinese” or “Israeli”) followed by either a Chinese or an Israeli face photograph and participants were asked to indicate whether the face matched the category label or not, in our study, the task was a simple race-categorization task in which participants only required a decision of whether a face presented in isolation was Chinese or Caucasian. Of course, the possible mechanism underlying the differential response patterns between category verification task and simple classification task should be investigated in the future. To our knowledge, the current experiment is the first report to directly investigate the association between SF and ORCA. It has been shown that the differential use of visual information while categorizing faces might be reflected in the SF scales. A LSF image retains the large-scale luminance variations and provides “a useful skeleton of the image” (Morrison and Schyns, 2001), which has been traditionally associated with the configuration of local parts (for a review, see Morrison and Schyns, 2001; Goffaux et al., 2005; Goffaux and Rossion, 2006). Therefore, the present findings confirmed that the configural computations by default play an important role for ORCA while categorizing an own-race face but not another-race face. Similarly, Zhao and Bentin (2011) found that the ORCA was significantly larger for distorted faces than for the normally configured faces, regardless of Chinese or Israeli participants.

In line with previous studies (e.g., Harel and Bentin, 2009), we also found that race categorization of faces was significantly slower and less accurate when the LSF was absent. However, we did not find the significant ORCA for HSF conditions, regardless of the race of the observers. As mentioned above, categorizing faces as other-race faces mainly relies on LSF information reflecting the global processing and hence, the HSF information is not the physiognomic information that is used to distinguish other-race faces. Based on the notion that high-frequencies are essential for capturing local details in the image (e.g., Goffaux and Rossion, 2006), local processing could not be necessary for categorizing other-race faces as well as the ORCA. However, Levin (2000) found the similar ORCA for upright and inverted faces, suggesting that a face’s race is best defined by its distinctive features. Actually, in Levin’s (2000) study the relationship between face inversion and ORCA should be concluded with caution because only one face template was used, in which the low-level visual information cannot be excluded. Moreover, one recent study found that the eyes are a much better cue at least for distinguishing between Chinese and Israelis (Zhao and Bentin, 2011). Although the differential race features may depend critically on the races that are compared (e.g., for African Americans the nose and mouth area often provides much more information) and the above findings cannot be easily generalized to any comparison of races, previous reports still appear to implicate that the local processing is also important to race classification of other-race faces (Levin, 2000; Zhao and Bentin, 2011). Indeed, HSF image preserves the sharp, fine-scale details of the image and associates with local parts of visual images, with different processing mechanism from isolated facial features such as the eyes. For instance, the N170 decreased in response to HSF faces but was larger in response to isolated eyes than full faces (e.g., Goffaux et al., 2003b; Itier et al., 2006). To this end, the relationship between HSF information and race categorization of faces need further investigation.

Before concluding, it should be noted that there was no significant effect of the observer’s race on face processing across spatial frequencies, that is, we did not find the cultural differences of face’s featural (reflected by HSF) and/or configural (reflected by LSF) processing. Indeed, the ORCA did not interact with race of participant, ruling out any account in terms of differences between the Caucasian and Chinese stimulus sets (see also, Zhao and Bentin, 2008). However, there was evidence that differential cultural backgrounds may play an important role in race perception of faces across races (e.g., Tanaka et al., 2004; Walker and Hewstone, 2006). For example, Tanaka et al found that Caucasian participants recognized own-race faces more holistically than Asian faces, whereas Asian participants demonstrated holistic recognition for both own-race and other-race faces; however, they considered that the differences in holistic recognition between Caucasian and Asian participants mirrored differences in their relative experience with own-race and other-race faces (Tanaka et al., 2004). Indeed, different from previous face processing within and across races using recognition memory task the present study investigated the race classification directly and hence, the task demands could be one of the sources of differential findings related to observers’ races (see also, Wiese, 2013). Interestingly, supporting the present findings, our previous study showed that, although the ORCA was significantly larger for distorted faces than for the normally configured faces, it was indeed not modulated by observers’ races (Zhao and Bentin, 2011). Although recent studies have provided abundant evidence for diversity in human cognition and behavior across cultures (e.g., Nisbett and Miyamoto, 2005) and that Westerners generally think in an analytical way, whereas East Asians generally think in a more holistic manner (e.g., Nisbett et al., 2001), the culture difference of processing faces with and across races need further investigation.

## Conclusion

Controlling the SF of face images, the present study investigated perceptual processing differences between own-race and other-race faces while faces are categorized by race. The own-race faces were classified faster and more accurately for BB faces than for both LSF and HSF faces, whereas, although other-race faces were classified less accurately for HSF than for either broad-band or low-frequency faces, there was no difference between BB and LSF conditions of other-race faces. Although it was not evident for HSF conditions, the ORCA was more evident for LSF than that for BB faces. Assuming that LSF scales are necessary for global/configural processing whereas HSF scales are necessary for detail/local processing, the present data indicate that global information is needed for subordinate race categorization of faces and that the automatic application of global/configural processing play an important role for ORCA while categorizing an own-race face. The present data provide new insights into cross-culture face perception.

## Author Contributions

GZ finished the experiment and the draft. JW, LZ wrote and revised this manuscipt. ZW finished the experiment and the data analysis.

## Conflict of Interest Statement

The authors declare that the research was conducted in the absence of any commercial or financial relationships that could be construed as a potential conflict of interest.

## Footnotes

1 www.adobe.com
